# Breaking the limits - multichromosomal structure of an early eudicot *Pulsatilla patens* mitogenome reveals extensive RNA-editing, longest repeats and chloroplast derived regions among sequenced land plant mitogenomes

**DOI:** 10.1186/s12870-022-03492-1

**Published:** 2022-03-09

**Authors:** Kamil Szandar, Katarzyna Krawczyk, Kamil Myszczyński, Monika Ślipiko, Jakub Sawicki, Monika Szczecińska

**Affiliations:** 1 Department of Botany and Nature Protection, University of Warmia and Mazury in Olsztyn, Plac Łódzki 1, 10-727 Olsztyn, Poland; 2 Laboratory of Translational Oncology, Intercollegiate Faculty of Biotechnology, University of Gdańsk and Medical University of Gdańsk, Dębinki 1, 80-211 Gdańsk, Poland; 3 Department of Ecology and Environmental Protection, University of Warmia and Mazury in Olsztyn, Plac Łódzki 3, 10- 727 Olsztyn, Poland

**Keywords:** *Pulsatilla*, Mitogenome, RNA-editing, Ranunculales, Intracellular transfer, Phylogeny

## Abstract

**Background:**

The mitogenomes of vascular plants are one of the most structurally diverse molecules. In the present study we characterize mitogenomes of a rare and endangered species *Pulsatilla patens*. We investigated the gene content and its RNA editing potential, repeats distribution and plastid derived sequences.

**Results:**

The mitogenome structure of early divergent eudicot, endangered *Pulsatilla patens* does not support the master chromosome hypothesis, revealing the presence of three linear chromosomes of total length 986 613 bp. The molecules are shaped by the presence of extremely long, exceeding 87 kbp repeats and multiple chloroplast-derived regions including nearly complete inverted repeat. Since the plastid IR content of Ranunculales is very characteristic, the incorporation into mitogenome could be explained rather by intracellular transfer than mitochondrial HGT. The mitogenome contains almost a complete set of genes known from other vascular plants with exception of *rps*10 and *sdh*3, the latter being present but pseudogenized. Analysis of long ORFs enabled the identification of genes which are rarely present in plant mitogenomes, including RNA and DNA polymerases, albeit their presence even at species level is variable. Mitochondrial transcripts of *P. patens* were edited with a high frequency, which exceeded the level known in other analyzed angiosperms, despite the strict qualification criteria of counting the editing events and taking into analysis generally less frequently edited leaf transcriptome. The total number of edited sites was 902 and *nad*4 was identified as the most heavily edited gene with 65 C to U changes. Non-canonical, reverse U to C editing was not detected. Comparative analysis of mitochondrial genes of three *Pulsatilla* species revealed a level of variation comparable to chloroplast CDS dataset and much higher infrageneric differentiation than in other known angiosperm genera. The variation found in CDS of mitochondrial genes is comparable to values found among *Pulsatilla* plastomes. Despite the complicated mitogenome structure, 14 single copy regions of 329 kbp, not splitted by repeats or plastid-derived sequences (MTPT), revealed the potential for phylogenetic, phylogeographic and population genetics studies by revealing intra- and interspecific collinearity.

**Conclusions:**

This study provides valuable new information about mitochondrial genome of early divergent eudicots, *Pulsatilla patens*, revealed multi-chromosomal structure and shed new light on mitogenomics of early eudicots.

**Supplementary Information:**

The online version contains supplementary material available at 10.1186/s12870-022-03492-1.

## Background

The mitogenomes of vascular plants are one of the most structurally diverse molecules despite generally stable gene content. After the divergence of evolutionary lineages of bryophytes and early vascular plants, the mitogenomes of the latter started to expand their intergenic regions [1]. Most of the structural variation in flowering plant mitogenomes are related to the presence of large repeats which enable homologous recombinations. In addition to the large, frequently recombining repeats, there are often smaller repeated sequences in the size lower than 1 kbp [2]. The frequency of the recombination appears to be positively correlated with the length of repeats [3, 4].

The size variation of angiosperm mitogenomes can be spectacular even between closely related taxa. Beside duplication of large parts of mitogenomes, the size expansion can be achieved by uptaking foreign sequences from plastid and nucleus or even extrinsic mitochondrial DNA via horizontal transfer [5]. The plastid-derived regions contribute 1 up to 10% of the mitochondrial genome size in vascular plants, however the majority of transferred genes were non-functional with a few exceptions of tRNA genes [6].

Unlike in non-flowering plants [1, 7, 8], the mitochondrial genome is present under different forms, not only circular one [9].

In recent years several comparative analyzes between closely related species were conducted, often revealing variation in structure and gene content [7, 9, 10], but the intraspecific variation was not the subject of many studies [11]. Due to mostly maternal inheritance mitogenomes may be an important source of evolutionary information, providing new insights into plant phylogeography and population genetics. However, frequent changes of mitogenome structures and assumed lower evolutionary rate compared to plastomes do not make mitochondrial genomes a first choice for studies on phylogeny and phylogeography, with exception of early land plant, bryophytes, which mitogenomes are rather evolutionarily stable [1, 7, 8] with few exceptions [12].

Modern technologies including long-read sequencing enabled obtaining sequences of many mitogenomes, but most of them belong to model or economically important and well-studied species [9, 13]. Both methods of long-read sequencing require high quality, high molecular weight DNA, which is sometimes difficult to obtain for species of limited tissue availability. In this study we employed PacBio and Oxford Nanopore Technologies (ONT) sequencing combined with DNA and RNA short-read sequencing to characterize mitogenome of a rare and endangered species *Pulsatilla patens*, belonging to Ranunculales order, which is basal for flowering plants although still poorly explored in terms of mitogenomics. In the present study we investigated the gene content and its RNA editing potential, repeats distribution and plastid-derived sequences. We also tried to answer the question if mtDNA can be a source of evolutionary information in phylogeography and conservation of endangered species.

Assuming the structural dynamics of mitogenomes, the application of complete genomic sequence in microevolutionary studies of vascular plants may be problematic due to relatively difficult assembly as well as a proper interpretation of recombination events. However, in this study we tried to identify and validate single copy mitogenomic regions, which can serve as a potential resource for population scale studies using mtDNA information.

## Results & discussion

### Sequencing results

The initial assembly strategy assumed using only pair-end (PE) and PacBio long reads seemed to be able to overcome the problems with plastid-derived regions and long repeats sequenced plant mitogenomes not exceeding 1-10 kbp range [2]. However, sequencing results obtained with PacBio (2 Gbp) revealed mitochondrial reads up to 2 kbp long and coverage 4-10x, which only resolved part of issues related with long repeats and plastid-derived regions. Better results were obtained with the nanopore sequencing, where 24-hour runs allowed to obtain over 8 Gbp of data. This resulted in 60x coverage of single copy mitochondrial regions and enabled proper orientation of repeat-flanking contigs.

Mapping PE reads onto assembled mitogenome revealed mean 531x coverage for single copy regions and relevantly multiplied for repeat regions (up to 5,000 x coverage).

### Mitogenome structure

**Fig. 1:**
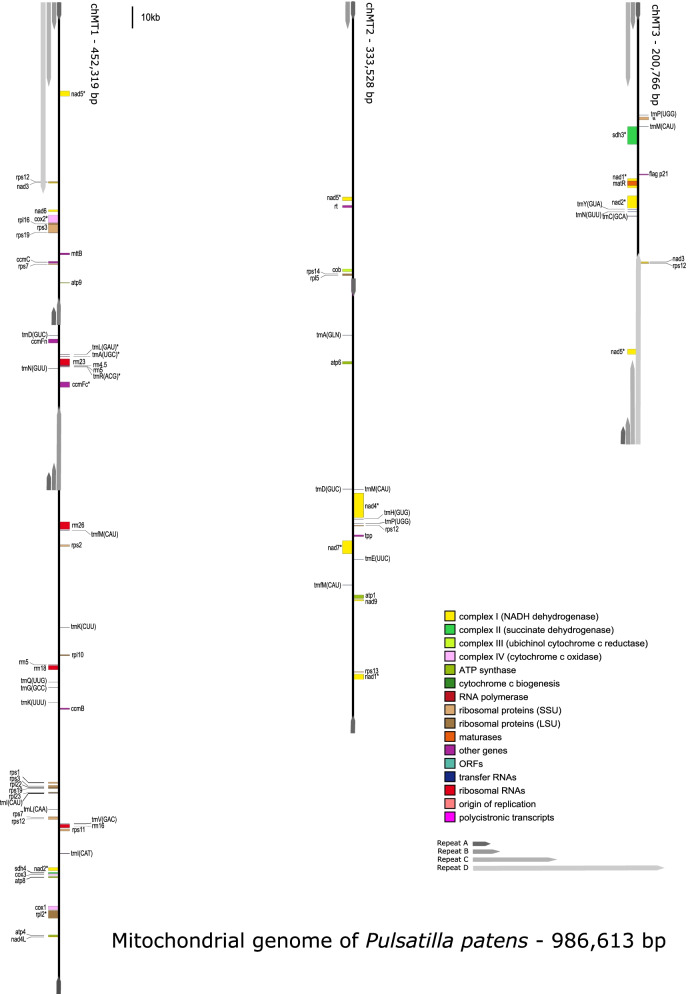
The mitogenome map of *Pulsatilla patens* spliced over three chromosomes. Asterix (*) indicate genes containing introns

Obtained results do not support the master chromosome hypothesis neither in circular nor linear form. Hybrid assembly using ONT and Illumina reads enabled to obtain three mitochondrial contigs (named chromosomes chMt1-chMt3) flanked by overlapping repeats A - D, which were also found inside chromosomes chMt1 and chMt2 (Fig. 1).

The main role in shaping the structure of *Pulsatilla* mitogenome play repeats up to 87 kbp long (Fig. 1). The longest of them, Repeat D is located at the beginning of chromosome chMt1 and the opposite end of chromosome chMt2. This repeat also contains remaining long repeats including almost 39 kbp long Repeat C, 13 kbp Repeat B and 8 kbp long Repat A (Fig. 1).

The Repeat C is flanking the end of chromosome chM3t and appears also inside chromosome chMt1. The end of chromosome chMt2 is flanked by Repeat B, which is also an internal part of chromosome chMt1, while remaining ends of chromosomes are flanked by Repeat A.

In the case of *Pulsatilla patens* the linearity of chromosomes chMt1 and chMt2, which contain large repeats A, B and C was supported only by six and eleven nanopore reads respectively, however, the junctions of single copy regions and large repeats were positively validated by long-range PCR amplification. On the other hand, PCR primers complementary to single copy regions closest to repeats present at chromosomes’ ends did not allow amplification of any detectable product, which does not/did not support circular master chromosome hypothesis. The fact that a single circular molecule is an oversimplified representation of the plant mitochondrial genomes and that they rather exist *in vivo* as a mix of circular, linear and branched forms is well documented [9, 14, 15]. However, a circular structure can usually be observed at the level of sequence assembly due to the presence of multiple repeats. In the case of *P. patens*, the presence of internal repeats did not allow the reconstruction of the master circle chromosome. The linearity of *P. patens* mitochondrial chromosomes is indirectly supported by unequal short and long reads coverage distribution along the main repeat. The hypothesis is also supported by the presence of features usually found in other species with linear single or multi-chromosomal mitochondrial genome: a terminal inverted repeat and the genes of RNA and DNA polymerases [16, 17]. The multi-chromosomal, linear mitogenomes were not previously reported in early eudicots, since *Aconitum*, *Anemone* and *Nelumbo* support the master circular chromosome hypothesis [18, 19].

Multi-chromosomal architecture of the mitochondrial genome was previously reported in several tracheophytes species and can form circular, linear or branched molecules. The diversification of mitogenome structures appear in the earliest tracheophyte lineages, while the evolutionarily older land plants, bryophytes exhibit single circular mitogenome molecules [20]. Starting with lycophytes, where single circular mitochondrial chromosome were confirmed only for *Phlegmariurus* [21–23], non-circular and multi-chromosomal mitogenomes are widely distributed among different fern, gymnosperm and angiosperm lineages. Two circular mitochondrial chromosomes were found in early divergent fern *Psilotum nudum*, while in *Ophioglossum californicum* master circle chromosome hypothesis was supported [24]. The sequenced gymnosperm mitogenomes of genera *Cycas*, *Ginkgo*, *Welwitschia* and *Taxus* could also be assembled into single circular molecules [25–27], but the larger, over 5 Mbp, mitogenomes of *Picea* species are described as multi-chromosomal and linear [28, 29]. The mitogenomes of angiosperms are structurally variable at genus or even species level. The largest and smallest known angiosperm mitogenomes belong to the species of *Silene*, which exist in single or multi-chromosomal, but usually circular forms [11]. Single and multi-chromosomal circular mitogenomes were reported for varieties of *Allium cepa* [30], and linear, branched or circular mitochondrial molecules were found in a single individual of *Lactuca sativa* [8]. Out of three mitochondrial chromosomes of *Solanum tuberosum* two could be assembled as circular, but the third exists in linear form [13].

The presence of master circular mitogenome structure can not be completely excluded. The assembly of *Chrysanthemum nakingense* mitogenome, using similar approach, confirmed the master chromosome structure relying only on four nanopore reads, but modules were not flanked by long inverted repeats [15], as it was in the case of *P. patens*.

### Gene content

The annotation of *Pulsatilla patens* mitogenome enabled the identification of a set of genes (rRNA, tRNA and CDS) that are typical for angiosperms (Table 1). According to classification [3] sequenced mitogenomes contain all “core” genes (*atp*1, *atp*4, *atp*6, *atp*8 *atp*9, *ccm*B, *ccm*C, *ccm*Fc, *ccm*Fn, *cob*, *cox*1-3, *mat*R, *mtt*B, *nad*1-7, *nad*9) and most of genes defined as “variable” (*rpl*1, *rpl*5, *rpl*10, *rpl*16, *rps*1-4, *rps*7, *rps*11-14, *rps*19, *sdh*4). These genes were also found in the three remaining *Pulsatilla* species including *P. alpina*, *P. pratensis* and *P. vernalis*. From the “variable” gene list only *rps*10 and *sdh*3 were not found in the *Pulsatilla* mitogenome. The fragments of *sdh*3 including exons 1 and 2 were found at chromosome chMt3, but they contain two internal stop codons and cover ca. 40% of functional genes. Both genes were not detected in RNA-seq analysis, so they were unlikely to be transferred to the nucleus. The presence of this gene pair (*rps*10 and *sdh*3) in plant mitogenomes seems to be correlated, since out of 18 cases of *rps*10 or *sdh*3 absence, in 15 cases both are missing [3]. Despite using deep sequenced libraries enabling nuclear genome coverage above 40X, the presence of these genes was not confirmed even in nuclear genomes.

**Table 1:** Gene content, localization and RNA editing of *Pulsatilla patens* mitogenome

Category	Gene	Location	Gene structure	RNA editing events	RNA editing frequency	CDS Length nc/aa	GC content
Complex 1	nad1	M1(e1);M2(e2-3);M3(e4-5)	e1;e2-i1-e3;e4-i2-e5	45	3,578,732,106	978	43,6
	nad2	M1(E1-E2); M3(E3-5)	e1-i1-e2;e3-i2-e4-i3-e5	43	2,810,457,516	1530	41
	nad3	ChM3	continous	19	5,322,128,852	357	42
	nad4	ChM1	e1-i1-e2-i2-e3-i3-e4	65CDS + 3 introns	4,36,827,957	1488	42,7
	nad4L	ChM1	continous	12	4,395,604,396	273	37,4
	nad5	ChM1 (E1-2), CHM2 (E3-E4)	e1-i1-e2,e3-i2-e4	39	1,937,406,855	2013	41,8
	nad6	ChM1	continous	17	2,791,461,412	609	41,2
	nad7	ChM2	e1-i1-e2-i2-e3-i3-e4-i4-e5	40CDS + 6 introns	3,410,059,676	1174	44,6
	nad9	ChM2	continous	11	1,919,720,768	573	42,9
Complex 2	sdh4	ChM1	continous	8	1,93,236,715	414	38,9
Complex 3	cob	ChM2	continous	22	1,842,546,064	1194	42,5
Complex 4	cox1	ChM1	continous	30	1,890,359,168	1587	44
	cox2	ChM1	e-i-e	18-cds, 1 intron	0,5,323,868,678	3381	49,4
	cox3	ChM1	continous	20	2,506,265,664	798	45,2
Complex 5	atp1	ChM1	continous	3	0,1,937,984,496	1548	44,3
	atp4	ChM1	continous	12	2,116,402,116	567	42,5
	atp6	ChM1	continous	25	3,267,973,856	765	38,7
	atp8	ChM1	continous	9	1,851,851,852	486	40.1
	atp9	ChM1	continous	1	0,4,444,444,444	225	37,3
Cytochrom c biogenesis	ccmC	ChM1	continous	40	5,376,344,086	744	44,4
	ccmB	ChM1	continous	42	6,763,285,024	621	42,7
	ccmFC	ChM1	e1-i-e2	25	1,081,782,778	2311	49,7
	ccmFN	ChM1	continous	34	1,960,784,314	1734	47,3
Ribosome large subunit	rpl2	ChM1	e1-i-e2	1	0,06,600,660,066	1515	51
	rpl5	ChM1	continous	10	1,792,114,695	558	44,1
	rpl10	ChM1	continous	6	1,19,760,479	501	42,9
	rpl16	ChM1	continous	10	2,096,436,059	477	45,3
Ribosome small subunit	rps1	ChM1	continous	5	0,7,278,020,378	687	42,8
	rps2	ChM1	continous	10	1,481,481,481	675	38,2
	rps3	ChM1	e1-i-e2	16	0,9,523,809,524	1680	43,2
	rps4	ChM3	continous	31	2,425,665,102	1278	39,3
	rps7	ChM1	continous	3	0,6,711,409,396	447	43
	rps11	ChM1	continous	4	0,8,888,888,889	450	44,2
	rps12	ChM1,ChM3	continous	11	2,91,005,291	378	45,2
	rps13	ChM2	continous	6	1,709,401,709	351	39,9
	rps14	ChM2	continous	2	0,7,575,757,576	262	40,2
	rps19	ChM1	continous	4	1,388,888,889	288	39,6
Translocation pathway	mttB	ChM1	continous	43	5,449,936,629	789	44,7
Maturases	matR	ChM3	continous	19	0,9,639,776,763	1971	52,5
HGT	DNA pol	chm1	continous	0	0	2850	38,7
	fasciclin-like	chm3	continous	0	0	396	50,8
	RNA pol	CHM1	continous	0	0	2154	33,6

The repeat units in the *P. patens* mitochondrion contain three protein coding genes: *nad*3, *nad*5 (exons 1-3) and *rps*12. All three genes are located in the Repeat D, over 48 kbp long duplicated region, resulting in two identical copies in the mitogenome. The third copy of *rps*12 is located in a single copy region with pairwise identity 81.9%. The RNA-seq analysis confirmed the expression of both *rps*12 variants. These genes are also duplicated or even triplicated in few known mitogenomes including *Cycas taitungensis* (AP009381), *Oryza minuta* (NC_029816) and *Vitis vinifera* (NC_012119). In the case of the mitogenome of *Daucus carota* (NC_017855), the *rps*12 gene is triplicated, but opposite to the *Pulsatilla* mitogenome, all three copies are identical. The partial duplication of *nad*5 gene was also described in mitogenomes of *Beta vulgaris* (BA000024), *Cynanchum auriculatum* (MH410146), *Rhazya stricta* (KJ485850) and *Tamarindus indica* (MN017227).

The *Pulsatilla patens* mitogenome contains two large ORFs encoding polymerases, which were recently reported in plant mitogenomes [9, 15]. The 2,850 bp long *Pp_DNA_pol_B* (encoding DNA polymerase) gene and 2,154 bp *Pp_RNA_pol* (encoding DNA-dependent RNA polymerase) are located on the chromosome chMt1.

The length of *Pp_DNA-pol_B* gene is similar in size to the previously reported *Ac_DNA_pol_B* gene (2,814 bp) found in *Actinidia chinensis* but the homolog of *Pp_RNA_pol* gene of this species is remarkably shorter amounting to 1,743 bp.

Mining available angiosperm mitogenomes towards polymerase encoding genes revealed their presence in over 50 accessions in the case of DNA polymerase and 18 accessions in the case of RNA polymerase assuming blastx identity threshold above >50%.

Both genes were reported from several mitogenomes, but the expression of them remained unconfirmed in most of the studies. Angiosperm mitochondrial genomes contain DNA derived from exons of the nuclear genome [31–34], but the examples of expression are rather rare [35]. The RNA-Seq analysis confirmed the presence of mRNA of polymerase coding genes in all three analysed samples. However, since availability of nuclear genomic resources of *P. patens* is limited to nuclear organizer regions (NOR) [36, 37], the nuclear origin of these transcripts can not be ruled out.

The presence of polymerase coding genes in the mitogenome seems to be limited to *P. patens*. Both genes are absent in the three other *Pulsatilla* species, including *P. alpina*, *P. pratensis* and *P. vernalis* as well as in more distant species of Ranunculales, *Anemone maxima* and *Aconitum kusnezoffii*. Moreover, among eight analysed specimens of *P. patens*, the presence of polymerase-like genes was not confirmed in individuals from populations STR6 and a115. The frequency of intraspecific presence of polymerase-like genes was not analysed in previous studies, but the lack of these genes in samples from southern populations [38] could indicate its recent transfer from nucleus.

Blasting longer orfs (>300 bp) also revealed other genes that are seldom or non-reported from mitogenomes of angiosperms. The 396 bp long orf on chMt3 chromosome was identified as fasciclin-like arabinogalactan protein 21 with the greatest similarity to sequences found in genomes of *Medicago truncata* (99% query coverage, 96.2% identity) and species of genus *Arachis* (97% query coverage and 83-85.4% identity). An unannotated gene was also found in the three chromosomes of *Cicer arietinum* (100% QC, 98.7 PI). However, all blastx (and blastn in case of *Cicer*) hits refer to nuclear genomes of species from Fabales, suggesting that this gene was recently acquired by intracellular sequence transfer from nucleus or was previously unannotated in known mitogenomes. Blasting against known mitochondrial genomes revealed the presence of this gene (97-100% query coverage and 99-95% identity) not only in mitogenomes of Fabales (*Lotus japonicus*, *Tamarindus indica*, *Styphnolobium japonicum* and *Medicago truncata*), but also in *Paraprenanthes diversifolia* and *Lactuca* species of Asterales. The phylogenetically scattered presence of this gene in angiosperm mitogenomes supports nuclear origin and its transfer to mitogenome independently in at least three angiosperm orders: Fabales, Asterales and reported for the first time Ranunculales.

The chromosome chMt1 of mitochondrial genome of *Pulsatilla patens* contains orf encoding RNA-dependent RNA polymerase (RdRP) and an expression of this gene was confirmed by RNA-seq analysis. The RdRP gene is recognized by its conserved protein domain family, pfam05919 and is presumably required for replication of mitoviruses [39] and was found in 40 out of 50 analysed plant mitogenomes [40]. The completeness of the RdRP gene in plant mitochondrial genomes varies from nearly complete versions of the RdRP to remnants barely detectable in sequence searches [40]. The RdRP gene found in the *Pulsatilla* mitogenome is 292 aa long and belongs to the longest among those found in plants. The further studies on *Pulsatilla* transcriptomes are required to confirm the presence of a complete mitovirus genome, which can be up to 4.4 kbp long [39].

The 328 aa long orf encoding reverse transcriptase with retrotransposon gag protein domain pfam03732 were found on chromosome chMt3 (blastx up to 90% of query coverage and >50% identity), however blasting against known plant mitogenomes did not provide any hits. The discovery of this gene encouraged us to perform LTR search in the assembled mitogenome, which are often considered as HGT vectors among nuclear and mitochondrial genomes [41]. Several orfs longer than 500 bp remain unidentified, as no significant hits were found in public databases, despite their expression end RNA editing events. However, the genomic resources of early eudicots are poorly explored.

### Phylomitogenomics relationships of *Pulsatilla patens*

**Fig. 2:**
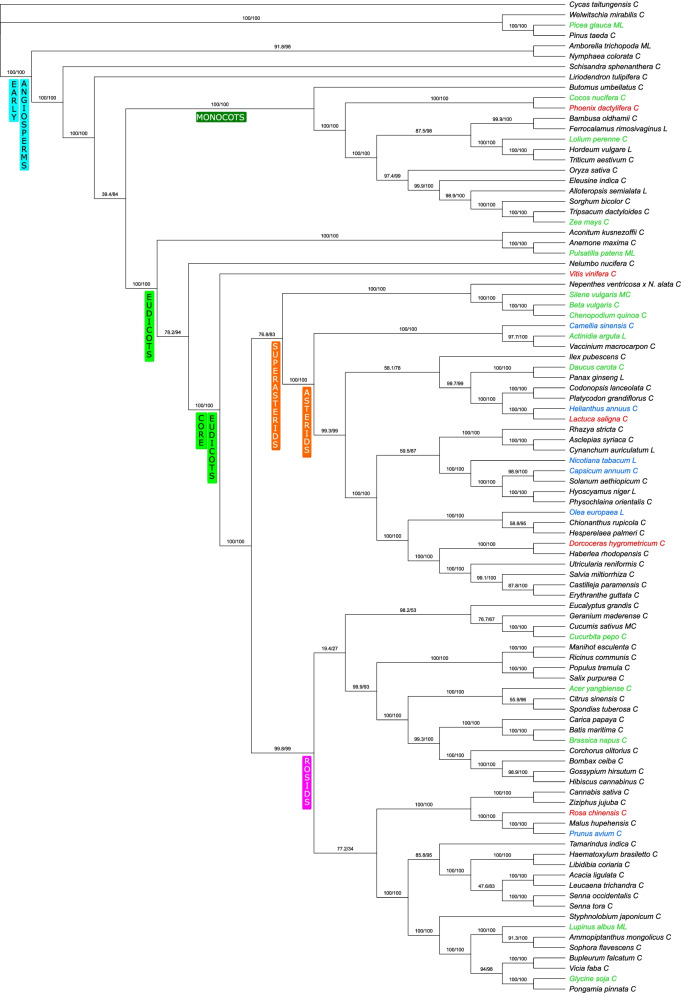
Phylogenetic tree based on protein-coding mitochondrial genes (green species names – genus contain both polymerase genes (*DNApol*, *RNApol*), blue species names – genus contain only DNA polymerase genes (*DNApol*), red species names – genus contain only RNA polymerase genes (*RNApol*); C - circular mitogenome, L – linear mitogenome, MC - multicircular mitogenome, ML – multilinear mitogenome)

The phylogenomic analyzes based on protein-coding mitochondrial genes resulted in trees with similar topologies (Fig. 2, Figure S1) and obtained topologies are congruent with previously published papers [42].

The phylogenetic analysis of 39 protein-coding mitochondrial genes reveals *Aconitum*, *Anemone* and *Pulsatilla* (Ranunculales) as early divergent eudicots with *Nelumbo nucifera* (Proteales) as its closest relative (Fig. 2). However, the phylogenomic analyses did not resolve Proteales-Ranunculales relationships with a maximal support. Previous studies using mitochondrial dataset [42], resolved *Nelumbo* as a basal for eudicots, however, it naturally did not include any member of Ranunculales. The phylogenetic position of *Pulsatilla patens* is congruent with plastome datasets, which resolved Ranunculales as an earlier divergent than Proteales [43]. Also, the analysis of 1,594 nuclear loci resolved Ranunculales as an older group than Proteales [44] and provided results congruent with chloroplast and mitochondrial data. The main aim of this analysis was the phylogenetic placement of mitogenomes containing polymerase genes (*DNApo*l, *RNApol*) which are scattered along the whole tree.

Phylogenetic relationships of mitochondrial *DNApol* and *RNApol* genes in many cases did not reflect the phylogenetic position of species (Figures S2-S3), suggesting it’s independent acquisition via horizontal gene transfer, which was reported several time in plant mitochondrial genomes [5, 45, 46]. The sequences of polymerase genes extracted from mitogenomes of closely related species usually group together, but in some clades even orders are mixed. The phylogenetic position of *Pp_RNA_pol* gene remained unresolved (Figure S3) and *Pp_DNA_pol* gene was clustered together with genes coming from evolutionary unrelated genera like *Theobroma*, *Actinidia* and *Prunus* (Figure S2).

### Repeats

Based on the currently known genomic resources, these large repeats seem to be limited to the genus *Pulsatilla*. The BLAST against known mitogenomes revealed only the presence of very short (up to 400 bp in case of *Ligustrum* sp.) parts of these repeats in other plant species. Each of three *P. patens* mitochondrial chromosomes is flanked with the same pair of inverted repeats, which seem to be unique among known mitogenomes. Chromosomes of *Solanum tuberosum* are also flanked with repeats, however in this case, the repeats were present in only two copies and their length ranged from 1,208 to 11,915 bp [13].

Beside mentioned large repeats, which shape the *P. patens* mitogenome structure, only two repeats longer than 1,000 bp were found. The repeats S1 (1,889 bp) and S2 (1,594 bp) were located on chMt2 and present in two copies. Additional two repeats shared among chromosomes fall within 500-1,000 bp range: repeats S3 (848 bp long) were found on chMt1 and chMt2 and repeat S4 (697 bp) were found on chMt2 and chMt3.

### Chloroplast-derived regions

DNA transfer from plastome to mitogenome is well documented in almost all vascular plant lineages [33, 47]. This transfer is usually not limited to a single gene, but in most cases it comprises a cluster of genes, which seems to diverge and fragment over evolutionary time [48, 49]. The *Pulsatilla patens*, which belongs to early divergent angiosperm order Ranunculales, reveals over 35 kbp of plastome derived regions in its mitogenome, called MTPT (plastid-derived mitochondrial DNA), which comprise 3.6% of total mitogenome length (Table S1). The pt-derived DNA is distributed among all the mt-chromosomes: five, three, and one transfer were found in chMt1, chMt2 and chMt3 respectively. Out of nine unique cp-mtDNA regions, five were transferred from chloroplast inverted repeat regions, including the longest, over 19 kbp. In total, almost the whole IR region (except gene *ycf*1) was transferred to mitochondrion in scattered form mainly over chMt1 with one part located on chMt2. The length of the longest MTPT is unusual, since the most of previously identified chloroplast-derived regions fall within 200-4,000 bp range [50] and the largest, 12.6 kbp long MTPT was found in *Zea mays* mitogenome [51]. In the other known mitogenome of Ranunculales, *Hepatica maxima*, the MTPT fragments are up tp 7.1 kbp long, with total length of 16 kbp [18] The presence of an almost complete IR region in *P. patens* mitogenome suggests that it was derived from single transfer that was subsequently split and partially duplicated by rearrangement. Similar scenario was described in *Silene conica*, where 35 kbp region was transferred in a single event, but later most of this MTPT was removed by a series of deletion events [51]. The mitogenome of *P. patens* contains 26.7% of plastome (excluding one copy of IR) which falls between *Cucumis melo* (22.7%) and *Bambusa oldhamii* (40.9%) Due to lack of mitogenomic data on early divergent eudicots it is difficult to infer the evolutionary significance of the amount of transferred cpDNA.

Among MTPT gene clusters identified by Wang et al. [49], three were not found in *P. patens* mitogenome: *atp*E-*rbc*L, *psb*E-*psb*F and *ycf*1-*trn*N. Another cluster transferred psaA-psaB, specific for eudicots, [49] was split among chromosomes chMt1 and chMt2 (Table S1). The *rpo*C1-*rpo*C2 cluster found on chromosome chMt3 was not specific for *Pulsatilla*, as blasting it against known plant mitogenomes confirmed its presence in six mitogenomes belonging to different orders: *Phoenix dactylifera* (Liliales), *Sapria himalayana* (Malpighiales), *Solanum aethlopicum* (Solanales), *Ziziphus jujuba* (Rosales), *Spondias tuberosa* (Sapindales) and *Ammopiptanthus mongolicus* (Fabales). Recent studies hypothesise that plastome sequences were initially acquired by intracellular gene transfer and then were transferred among plant lineages via mitochondrial horizontal gene transfer [50], however in the case of *Pulsatilla* mitogenome this scenario is rather unlikely. The longest MTPT (chloroplast transfer 4) contains 11 genes, which order is characteristic for all known *Pulsatilla* chloroplast genomes and intergenic spacer sequences are specific for this genus [36, 37, 52, 53]. Separation of MTPT from plastome reads with the whole genome sequencing approach could be challenging, especially while only short reads sequencing results are available, which could be mapped on both regions making them look like each other. Application of two long reads sequencing platform enabled presence of core mtDNA and MTPT in single reads, which confirmed the mitochondrial origin of MTPT which fall within the range of cpDNA variation at intraspecific level [36] suggesting it’s acquisition via intracellular gene transfer.

### RNA editing

According to previous studies RNA editing is obligatory for few mitochondrial genes by creating initiation or termination codons [13]. In the *Pulsatilla patens* mitochondrial genome, the C->U editing is required to create start codon in *nad*1 and stop codons in *atp*6, *rps*11, *ccm*FC, and *cox*2 genes.

The mitochondria of flowering plants usually have 300–750 sites that are subjected to RNA editing [54]. Despite considering only editing events with frequency above 0.5, the number of affected sites (907) is bigger than in *Solanum* [13] and *Populus* [55], where 799 and 355 editing events were detected respectively. High number of editing sites in CDS of *P. patens* mitogenome can not be explained by the type of the tissue used for RNA extraction, since leaf (as in case of our study) and root tissues are considered as less frequently edited than flower tissues [56]. The lower number of predicted RNA-editing sites was given for *Hepatica maxima* [18] due to presence of two genes missing in *P. patens* (*rpl*10 and *sdh*4). However, predicting-based estimation of RNA-editing sites usually overstates the amount of RNA-seq verified editing sites [57].

The number of observed edited sites varies from one in *rpl*2 up to 65 in *nad*4 (Table 1, Fig. S4). Considering gene category, the highest number of edited base pairs at assumed frequency was found in the genes of Complex 1 (*nad*) and Cytochrome C biogenesis (*ccm*), which is congruent with data obtained for *Populus* [55] as well as for early land plants [57]. In our study, the high number of editing sites can not be explained by possible false-positive results often observed in many surveys where DNA and RNA libraries were prepared from different individuals. In the case of genetically variable, widely distributed species C to T mutation in protein-coding regions could be identified as editing events, leading to false-positive identification of RNA-edited sites.

### Interspecific variation in protein- coding genes

The comparative analyses of *P. alpina*, *P. patens* and *P. pratensis* protein coding genes (CDS) revealed 50 SNPs (43 dN and 7 dS) and 23 (18 dN and 5 dS) differentiating *P. patens* from *P. alpina* and *P. pratensis* respectively (Table S2). Eight out of 38 compared genes did not reveal any interspecific variation (*nad*1, *nad*3, *nad*6, *nad*9, *cox*3, *ccm*C, *rpl*2, *mtt*B) and in the next seven genes only single SNPs were detected (*nad*4, *nad*4L, *nad*5, *nad*7, *cox*1, *ccm*B, *rps*12, *mat*R). The interspecific comparison revealed that the genes of complexes I-IV are least variable, while the most of interspecific variation is accumulated in the genes of Complex V and genes of small ribosomal subunit. Among the genes of Complex V the lowest variation was found in *atp*9 (only two synonymous mutations), while the remaining nucleotide substitution among *atp* genes varied from 7 (*atp*8) to 12 (*atp*1).

The genes belonging to other groups revealed low variation, except from *ccm*FC where 16 substitutions were found.

Most mutations found in protein-coding genes were substitutions, however the indels were also identified in five genes including 2aa indel in *atp*4, 5aa in *ccm*FC, 3aa in *rps*1, 4aa indel in *rps*3 and the largest, 30aa indel in *rps*4. With the single exception of *atp*4 (3 bp shorter CDS in *P. pratensis* than in *P. patens*), the rest of indels were found in *P. alpina*.

Mitochondrial genes are usually considered as slower evolving than chloroplast genes [58, 59], but the comparative analyses at genus level are limited to only a few genera.

The number of mutations of *Pulsatilla* mitochondrial CDS did not deviate from chloroplast CDS, especially when comparing *atp* and *rps* genes. Chloroplasts CDS of *atp*A, *atp*B, *atp*E and *atp*I revealed three, two, one and one substitution respectively, despite analysing six individuals from three *Pulsatilla* species [36]. Also, the genes coding for small ribosomal subunit were less variable, with only two substitutions in *rps*11 and *rps*15. With exception of the most variable *ycf*1 (39 substitutions), the numbers of substitutions in the remaining, relatively variable chloroplast CDS, ranged from five to seven [36].

The raw numbers of indels and substitutions could be biased by differences in gene length, therefore the pi nucleotide diversity is often used to estimate relative differences in the variation among genes. The pi values in the case of analysed *Pulsatilla* species (excluding mentioned above non-variable genes) varied from 0.0003 (*mat*R, *nad*5) to 0.0516 for *rps*4 (Table S3). Nine of the ten most variable genes comprised genes from Complex V and small ribosomal subunit (Table S4), which corresponded to the raw numbers of detected SNPs.

The mitochondrial protein-coding genes of *Pulsatilla* genus revealed relatively high variation in comparison to other vascular plants. Only eight SNPs were found among mitochondrial genes in the genus *Larix* [60]. On the other hand, the number of SNPs at species level in *Silene vulgaris* was much higher (144 SNPs), but they mostly concentrated in three genes: *atp*1, *atp*6 and *cox*1 [11]. However, the genus *Silene* is known for its extreme mitochondrial diversity, not only at substitution level but also in structure and gene content [11].

### Mitochondrial genomes as a resource for phylogenetic and phylogeography studies

The plastomes of Ranunculaceae are structurally variable [36, 61], but at the genus level the gene order seems to be conserved [36, 37, 52, 53]. Currently the data on sequenced mitogenomes of Ranunculaceae are restricted to three species, with different mitogenome structure, gene content and order. However, based on preliminary mappings of sequenced short-read libraries from *Pulsatilla alpina*, *P. vernalis* and *P. pratensis*, the selection of regions for phylogenetic and phylogeographic studies on generic level is quite challenging due to common presence of large repeats and MTPTs. Moreover, the linearity of preferred regions should be conserved among species. Eight single copy linear regions of minimum length 10 kbp with shared, identical gene order among analysed species were identified on chromosome chMt1 at 89-122 K, 149-160 K, 168-179 K, 223-252 K, 283-299 K, 303-333 K, 377-399 K, 409-424 K. Together 165 kbp of the total 452 kbp (36%) of chromosome chMt1 were suitable for low-level phylogenetic and phylogeographic studies. In the case of shorter chromosome chMt2, four regions meeting above criteria was found: 83-94 K, 101- 126 K, 214-276 K, 295-323 K, which consist 37% (126 K kbp) of total chMt2 length. At chromosome chMt3 only two phylogeny suitable regions were found between 49 and 67 K and 80-100 K, which consisted 19% (38 kbp) of total chMt3 length. The phylogenetic usage of mitogenomes in plant studies is mainly limited to CDS sequences, due to high evolutionary dynamics of molecule structure. The beyond-CDS mitogenomic phylogenetics is limited mainly to early land plants with a stable structure like liverworts [58] and mosses [7, 62, 63] and few studies on vascular plant taxa where both CDS and intron sequences were used [64]. The amount of shared mitogenomic clusters among *Pulsatilla* species seems to be high in comparison to genera like *Picea*, where only two shared clusters up to 9 kbp were found [29]. Mitochondrial conserved regions seem to have a great potential in phylogenetic studies, despite their taxa specific restrictions. As in the case of plastid genomes, they can provide better phylogenetic resolution than plastomes [65].

## Conclusions

Mitochondrial genome of early divergent eudicots, *Pulsatilla patens* revealed multi-chromosomal structure driven by extraordinary long repeats, and shed new light on mitogenomics of early eudicots. The presence of the longest, continuous MTPT with structure characteristic for *Pulsatilla* plastomes suggests its acquisition via intracellular transfer, not via mitochondrial HTG as recently hypothesised. The mitogenome sequences of *Anemone*, *Aconitum* and *Pulsatilla* confirm Ranunculales as earliest divergent eudicots.

## Methods

### Plant material and nucleic acids extraction

**Fig. 3:**
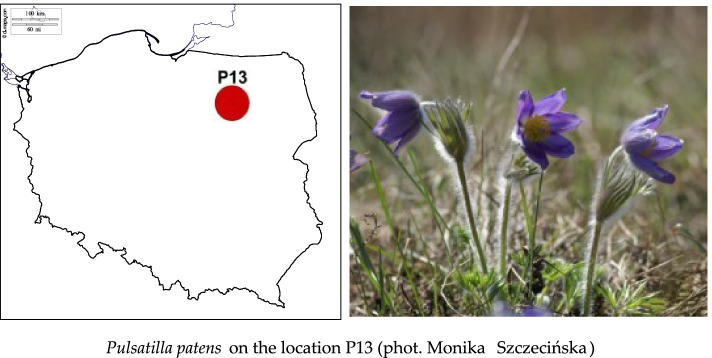
Location of sampled specimens of *Pulsatilla patens*

Plant material used in this study was collected from the Polish population of *Pulsatilla patens* (P13 - Rudne, Poland; 53^o^23’N, 21^o^35’E, Fig. 3) during previous population studies [38]. Formal identification of the plant material was performed by Monika Szczecińska. All plant material has been deposited in the Herbarium of Department of Botany and Nature Protection in Olsztyn (OLS) with specimen label: *Pulsatilla patens* P13 - Rudne.

The DNA and RNA was isolated from the leaves of the same individual. The mature leaves of the healthy plant during its flowering stage were collected and used for total RNA extraction immediately after collection using modified phenol/SDS method for plant RNA preparation [66]. Adequate quality and quantity of RNA samples were ensured by Bioanalyzer (Agilent) analysis. The RNA integrity value was measured using Bioanalyzer 2100 (Agilent Technologies, Santa Clara, California, USA). The purified total RNA was used for sequencing library preparation.

Total genomic DNA was extracted from fresh tissue immediately after collection. Ca 1 cm2 of cleaned leaf tissue was ground with silica beads in a MiniBead-Beater homogenizer for 50 s and subsequently processed following CTAB protocol [67].

DNA quantity was estimated using the Qubit fluorometer and Qubit™ dsDNA BR Assay Kit (Invitrogen, Carsbad, NM, USA). DNA quality was checked by electrophoresis in 0.5% agarose gel stained with Euryx Simple Safe (Eurx, Gdańsk, Poland). The extracted DNA prior to long-read sequencing was carefully examined and additionally cleaned using Genomic DNA Clean and Concentrator kit (Zymo, Irvine, USA).

### Genomic DNA Illumina sequencing

The genomic libraries for short-read sequencing were constructed with TruSeq DNA kit (Illumina, San Diego, CA, USA) and were sequenced using HiSeqX (Illumina) to generate 150 bp paired-end reads at Macrogen Inc. (Seoul, Korea) with 350 bp insert size between paired-ends.

### Nanopore sequencing

The long-read libraries were constructed using Ligation Sequencing Kit SQK-LSK109 (Oxford Nanopore Technologies) and NEBNext® Companion Module for Oxford Nanopore Technologies® Ligation Sequencing (New England Biolabs) according to manufacturer’s protocol and sequenced using MinION MK1B portable device (ONT) and R.9.4 Flow Cell (ONT). The Flow Cell was prepared for sequencing with Flow Cell Priming Kit EXP-FLP002 (ONT). Sequence reads were basecalled using high-accuracy guppy basecalling on MinKNOW platform.

### PacBio sequencing

The sample was prepared according to a guide for preparing SMRTbell template for sequencing on the PacBio Sequel System. The library preparation and sequencing were done by Macrogen Inc. (Seoul, Korea).

### Reads polishing

In order to use sequencing reads of the best quality the nanopore reads were polished using hybrid read error correction method. First, the Burrows-Wheeler Transform (BWT) of the short-read Illumina dataset was constructed using ropeBWT2 [68]. Next, FMLRC [69] was used to build FM-index and correct errors occurring within nanopore reads.

### RNA sequencing

Total RNA extraction was performed immediately after collection using a modified phenol/SDS method for plant RNA preparation [66]. Adequate RNA quality and quantity of RNA samples were ensured by Bioanalyzer (Agilent) analysis. The RNA integrity value was measured using Bioanalyzer 2100 (Agilent Technologies, Santa Clara, California, USA). The purified total RNA was used for sequencing library preparation. The extracted RNA was used for library construction using Truseq RNA kit with Ribo-Zero option (Illumina) and sequenced using Illumina NovaSeq 6000 platform by Macrogen Inc. (Seoul, Korea).

### Mitogenome assembly and annotation

To avoid extensions of chloroplast genome sequences, the complete plastid genomes were assembled using NOVOplasty v2.8. [70] with previously published *Pulsatilla patens* plastome as reference [36]. The unmapped reads generated from three platforms were used for contig assembly using SPAdes hybrid assembler. The obtained contigs were imported into Geneious Prime to identify contigs containing mitochondrial genes based on available plant mitogenomic resources in GenBank. Initial repeat analysis revealed presence of multiple repeats in 100-1,000 bp range and to avoid misassembly the nanopore and PacBio reads shorter than 2 kbp were removed from further analyses. The sequences containing mitochondrial genes were extended by mapping using Geneious mapper with five iterations with minimal overlap of 3,000 bp and overlap identity of 95%. This approach enabled assembly of six scaffolds containing all known mitochondrial genes. These sequences were flanked by large 8-87 kbp repeats. Careful examination of nanopore reads enabled the proper orientation of three flanking regions, reducing the number of scaffolds to three, which can not be assembled together due to opposite orientation of flanking repeats. The assembly was verified by mapping of PE reads with min overlap of 140 bp and identity of 99% which revealed ca. 600x coverage of single copy regions and proportionally higher at repeats and plastid derived regions.

Mitochondrial protein-coding genes were annotated using MITOFY web-based software [31]. The congruence with ORFs predicted by Geneious software (with 300 bp minimal length) were manually checked. Exonic-intronic boundaries were corrected using RNA-seq library reads. The ORFs identified within the intergenic spacer and longer than 300 bp length were blasted against the GenBank database using BLASTX. The rRNA and tRNA genes were identified using RNAmmer 1.2 [71] and tRNAscan-SE version 1.21 [72], respectively. The repeats were identified using RepeatFinder plugin for Geneious suite with minimum length set as 500 bp and up to 15% mismatches between repeats. The transposable elements were detected and classified using the LTR_Retriver package [73].

### Identification and confirmation of RNA editing sites

In order to predict C-to-U and U-to-C RNA editing sites, the PREPACT 3.12.0 (Universität Bonn, Bonn, Germany) [74] tool was used with the BLASTX mode and 0.001 e-value cut-off.

The RNA-seq reads obtained from the same individuals as DNA were used to confirm predicted RNA editing sites. The transcriptome and genome datasets were compared using RES-Scanner with default settings [75]. The editing frequency was calculated using a previously published approach [57].

### Phylogenetic analyses

The phylogenetic relationships were investigated using three datasets. The main dataset contains 29 protein-coding genes present in the most known angiosperm mitogenomes including: *atp*1, *atp*4, *atp*6, *atp*8-9, *ccm*B, *ccm*C, *ccm*FC,*ccm*FN, *cob*, *cox*1-3, *mat*R, *mtt*B, *nad*1-7, *nad*9, *rpl*2, *rpl*5, *rpl*10, *rpl*16, *rps*1-4, *rps*8, *rps*11-14, *rps*19 and *sdh*4. Next two datasets included RNA and DNA polymerase genes which were found in *P. patens* and other species mitogenomes.

Extracted protein-coding genes were aligned using MAFFT [76] and trees were calculated using IQ-Tree [77] under the model automatically selected by IQ-TREE (‘Auto’ option in IQ-TREE) for 5000 ultrafast [78] bootstraps, as well as the Shimodaira–Hasegawa–like approximate likelihood-ratio test [79]. Optimal evolutionary models for each gene were selected on the basis of BIC criterion calculated using Modeltest GTR+F+I+G4 [80–82].

### Variation detection

The sequences of *P. alpina* and *P. pratensis* mitochondrial genes were obtained by mapping short reads to *P. patens* mitochondrial genes dataset extracted from the assembled genome.

The SNPs were detected using Geneious Prime 2019 software (Biomatters, Auckland, New Zealand) with options: minimum coverage set to 20 and minimum variant frequency set to 0.8. The pi diversity values were obtained using Tassel 5.2.60 software [83].

## Supplementary Information

**Additional file 1:** **Figure S1.** Phylogenetic relationships based on amino acids dataset.

**Additional file 2:** **Figure S2.** Phylogeny on mitochondrial DNA polymerases.

**Additional file 3:** **Figure S3.** Phylogeny of mitochondrial RNA polymerases.

**Additional file 4:** **Figure S4.**  RNA editing of mitochondrial gene.

**Additional file 5:** **TableS1.** Chloroplast derived regions in mitochondrial DNA of *Pulsatilla patens*. Table S2. Comparative analyses of *P. alpina*, *P.patens* and *P. pratensis* protein coding genes. Table S3. Pi diversityof protein coding mitochondrial genes. Table S4. Gene contents of *Pulsatillapatens* and relatives mitogenomes.

## Data Availability

The mitogenome sequences are deposited in GenBank with accession numbers MZ420977, MZ420978, MZ420979 for chromosomes MtCh1, MtCh2 and MtChr3 respectively. Raw RNA-seq reads are deposited in SRA archive with their SRA data accessions numbers: SRR10230554, SRR10230555, SRR10230556, SRR10230557.

## Declarations

The study was in accordance with the institutional/national guidelines.
