# Supplementary figures and images for: Breaking the limits - multichromosomal structure of an early eudicot Pulsatilla patens mitogenome reveals extensive RNA-editing, longest repeats and chloroplast derived regions among sequenced land plant mitogenomes
Source: BMC Plant Biol. 2022 Mar 9;22:109. doi: 10.1186/s12870-022-03492-1 (PMC8905907; doi:10.1186/s12870-022-03492-1)

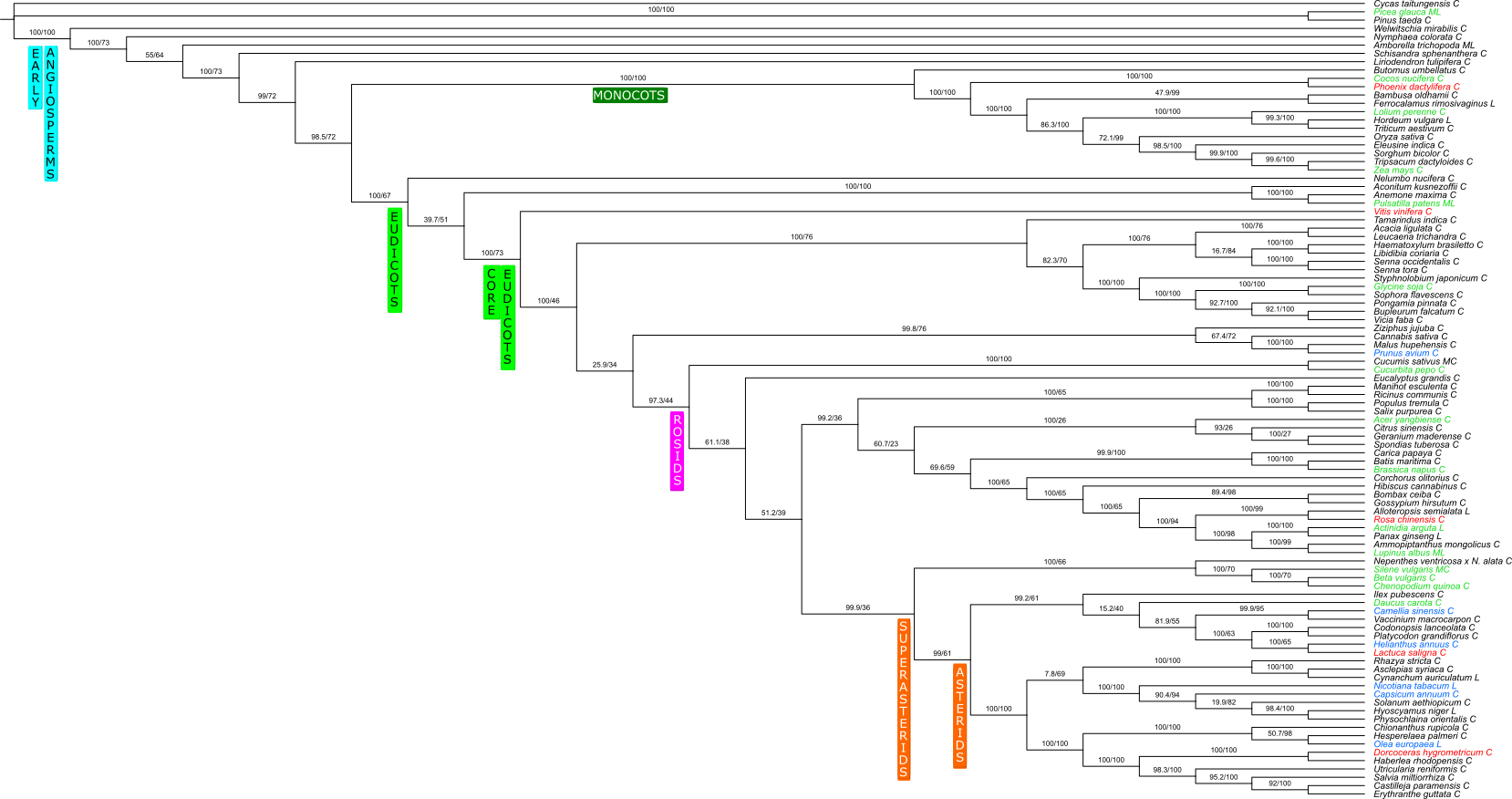

Supplement: Supplementary file 1 — Additional file 1: Figure S1. Phylogenetic relationships based on amino acids dataset. [file 12870_2022_3492_MOESM1_ESM.png]

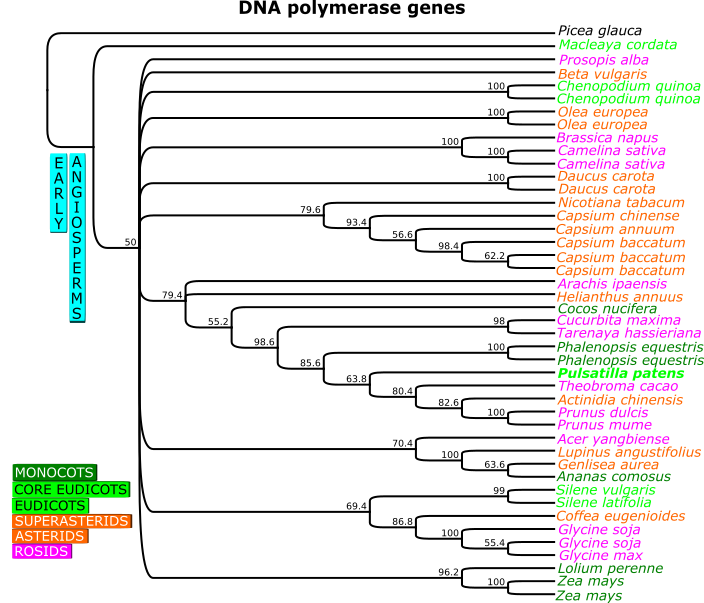

Supplement: Supplementary file 2 — Additional file 2: Figure S2. Phylogeny on mitochondrial DNA polymerases. [file 12870_2022_3492_MOESM2_ESM.png]

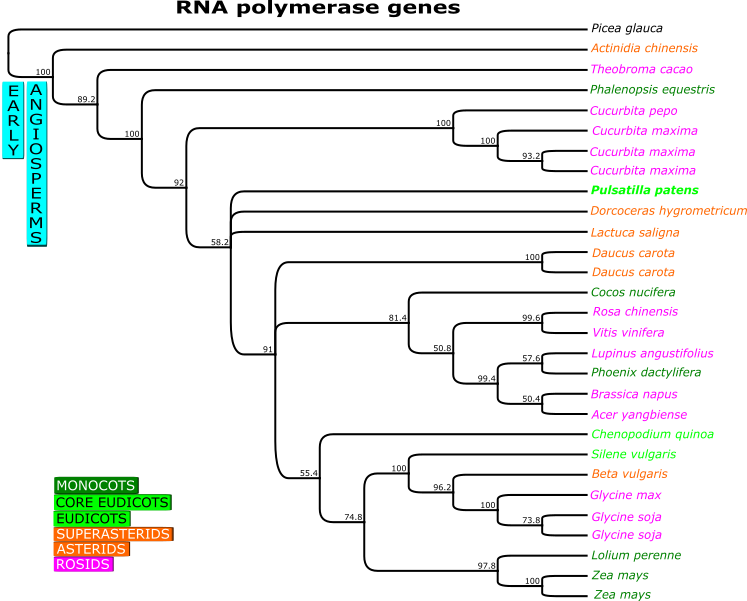

Supplement: Supplementary file 3 — Additional file 3: Figure S3. Phylogeny of mitochondrial RNA polymerases. [file 12870_2022_3492_MOESM3_ESM.png]

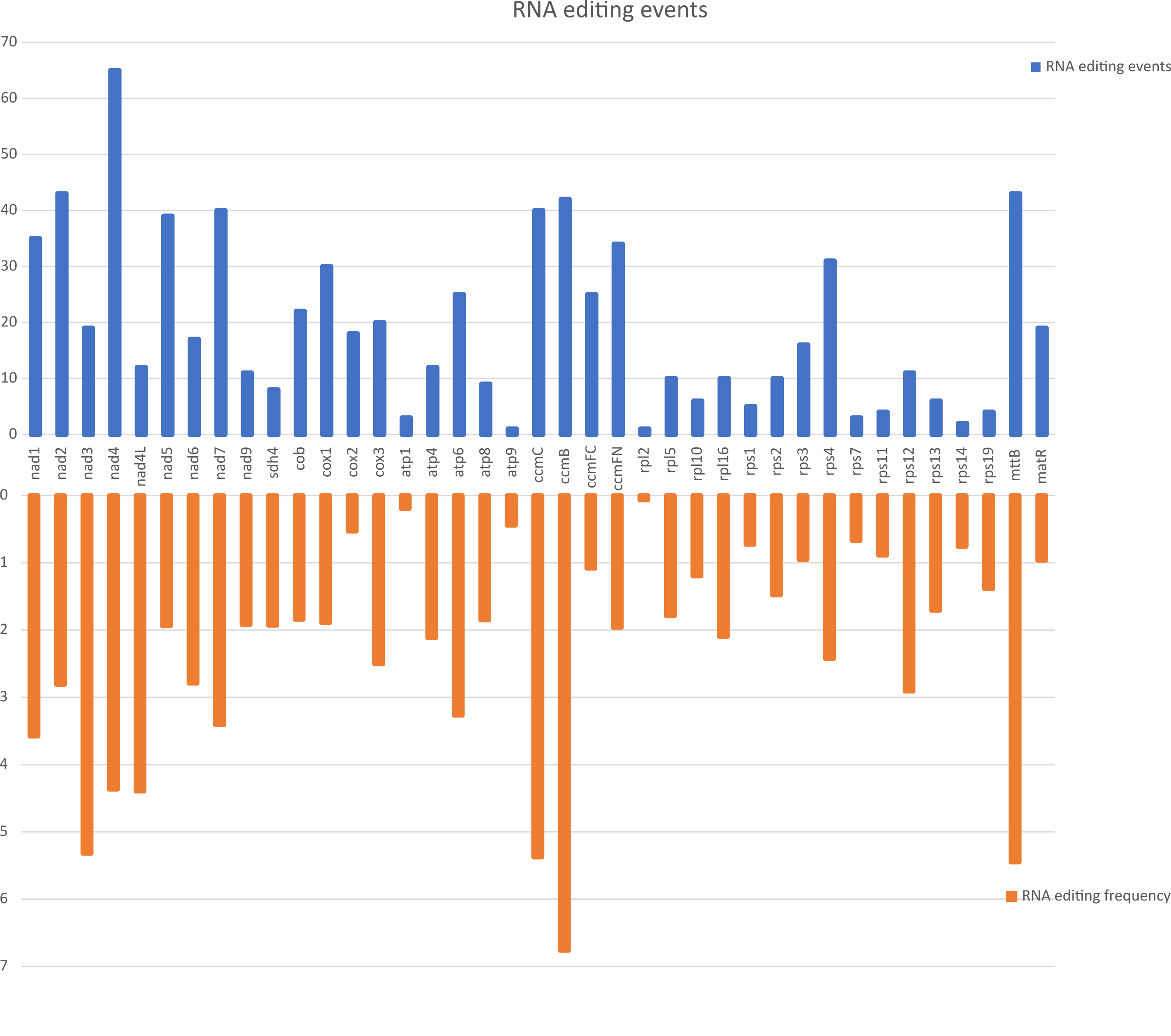

Supplement: Supplementary file 4 — Additional file 4: Figure S4. RNA editing of mitochondrial gene. [file 12870_2022_3492_MOESM4_ESM.png]
