# Supplementary material for: Breaking the limits - multichromosomal structure of an early eudicot Pulsatilla patens mitogenome reveals extensive RNA-editing, longest repeats and chloroplast derived regions among sequenced land plant mitogenomes
Source: BMC Plant Biol. 2022 Mar 9;22:109. doi: 10.1186/s12870-022-03492-1 (PMC8905907; doi:10.1186/s12870-022-03492-1)
Supplement: Supplementary file 5 — Additional file 5: TableS1. Chloroplast derived regions in mitochondrial DNA of Pulsatilla patens. Table S2. Comparative analyses of P. alpina, P.patens and P. pratensis protein coding genes. Table S3. Pi diversityof protein coding mitochondrial genes. Table S4. Gene contents of Pulsatillapatens and relatives mitogenomes. [file 12870_2022_3492_MOESM5_ESM.docx]

Table S1. Chloroplast derived regions in mitochondrial DNA of *Pulsatilla patens*.

| Annotation title | Choromosome | Location | Length (bp) | Plastome region | Chloroplast genes |
| --- | --- | --- | --- | --- | --- |
| chloroplast transfer 1 | chMt1 |  | 6519 | IR | trnN-trnR-rRNA5s-rRNA4.5S-rRNA23s-trnA-trnL(partial) |
| chloroplast transfer 2 | chMt1 |  | 643 | IR | rRNA16(partial) |
| chloroplast transfer 3 | chMt1 |  | 458 | IR | rRNA16(partial) |
| chloroplast transfer 4 | chMt1 |  | 19198 | IR | rps3-rpl22-rps19-rpl2-rpl23-trnL-ycf2-trnL-ndhB-rps7-trnV-rRNA16s (partial) |
| chloroplast transfer 5 | chMt1 |  | 635 | LSC | psaA(partial) |
| chloroplast transfer 6 | chMt2 |  | 1702 | LSC | psaA(partial)-psaB(partial) |
| chloroplast transfer 7 | chMt2 |  | 1396 | IR | rpl14(partial)-rpl16 |
| chloroplast transfer 8 | chMt2 |  | 1440 | LSC | psbC (partial) |
| chloroplast transfer 9 | chMt3 |  | 2952 | LSC | rpoC1(partial)-rpoC2(partial) |

Table S2. Comparative analyses of *P. alpina*, *P. patens* and *P. pratensis* protein coding genes

|  |  | P. alpina |  |  | P. pratensis |  |  |  | Pi |
| --- | --- | --- | --- | --- | --- | --- | --- | --- | --- |
| Category | Gene | dn | ds | remarks |  |  |  |  |  |
| Complex I | nad1 | 0 | 0 |  | 0 | 0 |  | 0 | 0 |
|  | nad2 | 0 | 0 |  | 0 | 0 |  | 0 | 0 |
|  | nad3 | 0 | 0 |  | 0 | 0 |  | 0 | 0 |
|  | nad4 | 1 | 0 |  | 0 | 0 |  | 1 | 0,00045 |
|  | nad4L | 1 | 0 |  | 0 | 0 |  | 1 | 0,00244 |
|  | nad5 | 0 | 1 |  | 0 | 0 |  | 1 | 0,00033 |
|  | nad6 | 0 | 0 |  | 0 | 0 |  | 0 | 0 |
|  | nad7 | 0 | 0 |  | 0 | 1 |  | 1 | 0,00057 |
|  | nad9 | 0 | 0 |  | 0 | 0 |  | 0 | 0 |
| Complex II | sdh4 | 3 | 0 |  | 0 | 0 |  | 3 | 0,00483 |
| Complex III | cob | 0 | 0 |  | 1 | 1 |  | 2 | 0,00112 |
| Compex IV | cox1 | 0 | 0 |  | 0 | 1 |  | 1 | 0,00042 |
|  | cox2 | 1 | 0 |  | 1 | 0 |  | 2 | 0,00868 |
|  | cox3 | 0 | 0 |  | 0 | 0 |  | 0 | 0 |
| Complex V | atp1 | 2 | 5 |  | 3 | 2 |  | 12 | 0,00517 |
|  | atp4 | 9 |  | 2aa del | 0 | 0 | 1 aa del | 9 | 0,01396 |
|  | atp6 | 9 | 1 |  | 0 | 0 |  | 10 | 0,01394 |
|  | atp8 | 3 | 2 |  | 1 | 1 |  | 7 | 0,0096 |
|  | atp9 | 0 | 2 |  | 0 | 0 |  | 2 | 0,01185 |
| Cytochrome C biogenesis | ccmC | 0 | 0 |  | 0 | 0 |  | 0 | 0 |
|  | ccmB | 0 | 0 |  | 0 | 1 |  | 1 | 0,00214 |
|  | ccmFC | 2 | 6 | 5aa del | 6 | 2 |  | 16 | 0,01094 |
|  | ccmFN | 2 | 1 |  | 0 | 0 |  | 3 | 0,00154 |
| Ribosome large subunit | rpl2 | 0 | 0 |  | 0 | 0 |  | 0 | 0 |
|  | rpl5 | 2 | 2 |  | 0 | 0 |  | 4 | 0,00478 |
|  | rpl10 | 2 | 0 |  | 2 | 0 |  | 4 | 0,00399 |
|  | rpl16 | 2 | 0 |  | 0 | 0 |  | 2 | 0,0028 |
| Ribosome small subunit | rps1 | 2 | 1 | 3aa del | 0 | 0 |  | 3 | 0,01164 |
|  | rps2 | 9 | 0 |  | 2 | 0 |  | 11 | 0,02469 |
|  | rps3 | 9 | 2 | 4aa ins | 5 | 1 |  | 17 | 0,01101 |
|  | rps4 | 3 | 0 | 30aa del | 2 | 4 |  | 9 | 0,05164 |
|  | rps7 | 1 | 0 |  | 1 | 0 |  | 2 | 0,00149 |
|  | rps11 | 4 | 0 |  | 1 | 0 |  | 5 | 0,00593 |
|  | rps12 | 0 | 0 |  | 0 | 0 |  | 0 | 0 |
|  | rps13 | 1 | 1 |  | 1 | 0 |  | 3 | 0,0057 |
|  | rps14 | 3 | 1 |  | 3 | 0 |  | 7 | 0,02778 |
|  | rps19 | 3 | 0 |  | 1 | 0 |  | 4 | 0,00926 |
| Translocation pathway | mttB | 0 | 0 |  | 0 | 0 |  | 0 | 0 |
| Maturases | matR | 1 | 0 |  | 0 | 0 |  | 1 | 0,00034 |
| HGT | DNA pol |  |  |  |  |  |  | 0 |  |
|  | fasciclin-like | loss |  |  | 0 | 0 |  | 0 |  |
|  | RNA pol |  |  |  |  |  |  |  |  |
|  |  |  |  |  |  |  |  |  |  |
|  |  |  |  |  |  |  |  |  |  |
|  |  | 42 | 7 | 0 | 18 | 5 |  |  |  |

Table S3. Pi diversity of protein coding mitochondrial genes.

| Gene | Pi diversity |
| --- | --- |
| rps4 | 0,0516 |
| rps14 | 0,0278 |
| rps2 | 0,0247 |
| atp4 | 0,0140 |
| atp6 | 0,0139 |
| atp9 | 0,0119 |
| rps1 | 0,0116 |
| rps3 | 0,0110 |
| ccmFC | 0,0109 |
| atp8 | 0,0096 |
| rps19 | 0,0093 |
| cox2 | 0,0087 |
| rps11 | 0,0059 |
| rps13 | 0,0057 |
| atp1 | 0,0052 |
| sdh4 | 0,0048 |
| rpl5 | 0,0048 |
| rpl10 | 0,0040 |
| rpl16 | 0,0028 |
| nad4L | 0,0024 |
| ccmB | 0,0021 |
| ccmFN | 0,0015 |
| rps7 | 0,0015 |
| cob | 0,0011 |
| nad7 | 0,0006 |
| nad4 | 0,0005 |
| cox1 | 0,0004 |
| matR | 0,0003 |
| nad5 | 0,0003 |
| nad1 | 0 |
| nad2 | 0 |
| nad3 | 0 |
| nad6 | 0 |
| nad9 | 0 |
| cox3 | 0 |
| ccmC | 0 |
| rpl2 | 0 |
| rps12 | 0 |
| mttB | 0 |

Table S4. Gene contents of *Pulsatilla patens* and relatives mitogenomes

|  | **Category** | ***Pulsatilla patens*** | ***Anemone eriantha*** | ***Anemone chinensis*** |
| --- | --- | --- | --- | --- |
| Native genes | Complex Ⅰ | *nad1, nad2, nad3, nad4, nad4L, nad5, nad6, nad7, nad9* | *nad1, nad2, nad3, nad4, nad4L, nad5, nad6, nad7, nad9* | *nad1b, nad2, nad3, nad4, nad4L, nad5, nad6, nad7, nad9* |
|  | Complex Ⅱ | *sdh4* | *sdh3, sdh4* | *sdh3, sdh4* |
|  | Complex Ⅲ | *cob* | *cob* | *cob* |
|  | Complex Ⅳ | *cox1, cox2, cox3* | *cox1, cox2, cox3* | *cox1, cox2, cox3* |
|  | Complex Ⅴ | *atp1, atp4, atp6, atp8, atp9* | *atp1, atp4, atp6, atp8, atp9* | *atp1, atp4, atp6a, atp8, atp9* |
|  | Cytochrome c biogenesis | *ccmB, ccmC, ccmFc, ccmFN* | *ccmB, ccmC, ccmFc, ccmFN* | *ccmB, ccmC, ccmFca, ccmFN* |
|  | Ribosome large subunit | *rpl2, rpl5, rpl10, rpl16* | *rpl2, rpl5, rpl10, rpl16* | *rpl2, rpl5, rpl10, rpl16* |
|  | Ribosome small subunit | *rps1, rps3, rps4, rps7, rps11, rps12, rps13, rps14, rps19* | *rps1, rps3, rps4, rps7, rps10, rps12, rps13, rps14, rps19* | *rps1, rps3, rps4, rps7, rps10, rps12, rps13a, rps14, rps19* |
|  | Translocation pathway | *mttB* | *mttB* | *mttB* |
|  | Group II introns | *ccmFci829, cox2i691, nad1i394c, nad1i477, nad1i669c, nad1i728, nad2i156, nad2i542c, nad2i709, nad2i1282, nad4i461, nad4i1399, nad5i230, nad5i1455c, nad5i1477c, nad5i1872, nad7i140, nad7i209, nad7i676, nad7i917, rpl2i846, rps3i74, rps10i235* | *ccmFci829, cox2i691, nad1i394c, nad1i477, nad1i669c, nad1i728, nad2i156, nad2i542c, nad2i709, nad2i1282, nad4i461, nad4i1399, nad5i230, nad5i1455c, nad5i1477c, nad5i1872, nad7i140, nad7i209, nad7i676, nad7i917, rpl2i846, rps3i74, rps10i235* | *ccmFci829a, cox2i691, nad1i394c, nad1i477a, nad1i669c, nad1i728, nad2i156, nad2i542c, nad2i709, nad2i1282, nad4i461, nad4i1399, nad5i230, nad5i1455c, nad5i1477c, nad5i1872, nad7i140, nad7i209, nad7i676, nad7i917, rpl2i846, rps3i74, rps10i235* |
|  | Intronic ORF | *matR* | *matR* | *matR* |
|  | rRNA genes | *rrn5, rrn18, rrn26* | *rrn5, rrn18, rrn26* | *rrn5, rrn18, rrn26* |
|  | tRNA genes | *tRNA-Asn,tRNA-Asp,tRNA-Cys,tRNA-Gln,tRNA-Glu,tRNA-Gly,tRNA-His,tRNA-Ile,tRNA-Leu(2),tRNA-Lys(2),tRNA-Met(4),tRNA-Phe,tRNA-Pro(2),tRNA-Ser(2),tRNA-Tyr* | *tRNA-Asn,tRNA-Asp,tRNA-Cys,tRNA-Gln,tRNA-Glu,tRNA-Gly,tRNA-His,tRNA-Ile,tRNA-Leu(2),tRNA-Lys,tRNA-Met(4),tRNA-Phe,tRNA-Pro(2),tRNA-Ser(2),tRNA-Tyr* | *tRNA-Asn,tRNA-Aspa,tRNA-Cys,tRNA-Gln,tRNA-Glu,tRNA-Gly,tRNA-His,tRNA-Ile,tRNA-Leu(3b),tRNA-Lys(2),tRNA-Met(4b),tRNA-Phe,tRNA-Pro(2),tRNA-Ser(2),tRNA-Tyr* |
|  | Hypothetical genes | *-* | *-* | *DNA polymerase-like, DNA-dependent RNA polymerase* |
| Plastid-derived genes | Genes with intact ORFs | *petG_cp, psbJ_cp, petL_cp, rpoC1_cp, rps7_cp* | *petG_cp, psbJ_cp, petL_cp* | *ndhC_cpa, ndhJ_cpa, ndhK_cpa, petG_cp, psbJ_cp, petL_cp, rbcL_cp, rpl2_cp, rpl23_cp, rps4_cpa, rps7_cp, rps19_cp* |
|  | tRNA genes | *tRNA-Val_cp, tRNA-Trp_cp* | *tRNA-Trp_cp* | *tNRA-Thr_cpa, tRNA-His_cp, tRNA-Met_cp, tRNA-Phe_cpa, tRNA-Ser_cpa, tRNA-Trp_cp, tRNA-Val_cp* |
|  | rRNA genes | *rrn16_cp* | *-* | *rrn16_cp* |
| HGT genes | Genes with intact ORFs | *rps2* | *rps2Ψ* | *rps2Ψ* |
